# Intestinal Ischemia: Unusual but Fearsome Complication of COVID-19 Infection

**DOI:** 10.3390/biomedicines10051010

**Published:** 2022-04-27

**Authors:** Silvia Strambi, Agnese Proietti, Christian Galatioto, Federico Coccolini, Camilla Cremonini, Serena Musetti, Fulvio Basolo, Massimo Chiarugi, Dario Tartaglia

**Affiliations:** 1 General, Emergency and Trauma Surgery Unit, Pisa University Hospital, 56124 Pisa, Italy; federico.coccolini@unipi.it (F.C.); camilla.cremonini@phd.unipi.it (C.C.); serena.musetti@phd.unipi.it (S.M.); massimo.chiarugi@unipi.it (M.C.); dario.tartaglia@unipi.it (D.T.); 2 Anatomic Pathology Section, Department of Surgical, Medical, Molecular Pathology, and Critical Area, Pisa University Hospital, 56124 Pisa, Italy; a.proietti@ao-pisa.toscana.it (A.P.); fulvio.basolo@unipi.it (F.B.); 3 General Surgery Unit, Ospedali Riuniti di Livorno, 57124 Livorno, Italy; christian.galatioto@uslnordovest.toscana.it

**Keywords:** bowel ischemia, COVID-19, SARS-CoV-2, thromboembolism

## Abstract

The pathophysiology of gastrointestinal damage in coronavirus disease (COVID-19) is probably multifactorial. It is not clear whether the etiology of intestinal ischemia may be directly related to viral replication or may result from hyper-coagulability following SARS-CoV-2 infection.To confirm a pathogenic role of COVID-19, we retrospectively investigated the presence of SARS-CoV-2 virus in the ischemic bowel of five COVID-19 patients undergoing emergency surgery for intestinal ischemia in the period of March 2020–May 2021. Immunohistochemical positivity with weak intensity was observed in four out of five cases, but only one case was strongly positive both at immunohistochemistry and at molecular analysis. The histological alterations in the intestinal tissue samples showed similarity with the well-known alterations described in typical targetorgans of the virus (e.g., the lung). This observation suggests a similar mechanism of action of the virus. Further larger studies are, thus, required to confirm this preliminary finding. Clinicians should carefully monitor all COVID-19 patients for the possible presence of a SARS-CoV-2 intestinal infection, a potential cause of ischemia and bowel perforation.

## 1. Introduction

Severe acute respiratory syndrome coronavirus 2 (SARS-CoV-2), responsible for the coronavirus disease 2019 (COVID-19) has rapidly spread around the world, officially since December 2019 [1]. At the onset of the COVID-19 disease, the most common symptoms are fever, cough, fatigue, myalgia, and also dyspnea, since the primary target of SARS-CoV-2 is the lung. However, COVID-19 is a systemic disease that can affect multiple organs in the body.

The incidence of gastrointestinal manifestations such as nausea, vomiting, and diarrhea has been reported in up to 39% of SARS-CoV-2 infected patients [2]. Gastrointestinal symptoms may be associated with a longer illness duration but have not been linked to increased mortality [3,4]. Gastrointestinal symptoms at presentation were the predominant complaint in 20% of patients and the only presenting symptoms of COVID-19 in 14% [5].

A multifactorial origin has been proposed for the gastrointestinal damage in COVID-19. Several years ago, Li et al. [6] demonstrated that SARS-CoV uses host angiotensin-converting enzyme 2 (ACE2) as the receptor for fusion of viral and host membranes. The same mechanism was proposed for SARS-CoV-2; nevertheless, ACE2 levels in the gastrointestinal tract have not been demonstrated to be elevated, and so the role of the co-receptor transmembrane serine protease 2 (TMPRSS2) was postulated to allow the virus to infect the epithelial cells and replicate on the luminal side of the small intestine; the gastric, duodenal, and rectal epithelial cells; and the glandular enterocytes [7,8]. Alternatively, the virus could spread through blood dissemination from the respiratory tract to other organs, such as the gut. In fact, microvascular injury (ischemic enteritis, patchy bowel necrosis, presence of thrombi, and perivascular inflammation) was observed in the submucosal arterioles of the small intestine from patients with COVID-19 [9,10]. Finally, the hypothesis of inflammation-mediated tissue damage was proposed and supported by the observation of infiltrating plasma cells and lymphocytes and of interstitial edema in the lamina propria of the stomach, duodenum, and rectum of patients [11].

We report a case series of COVID-19 patients presenting intestinal ischemia with a focused review of the literature.

## 2. Materials and Methods

Twentycases of COVID-19 patients undergoing an operative procedure in the period of March 2020–May 2021 at the Emergency Surgery and Trauma Center Department of the University of Pisa (Italy) were included in a prospective database. All patients had positive nasopharyngeal swab and pulmonary involvement to some extent. They granted informed consent and were treated with the usual institutional standard of care. Those presenting an intestinal ischemia were retrospectively analyzed in depth. The demographic features and operative details of the study cohort are summarized in Results. 

The presence of SARS-CoV-2 was investigated with anti-SARS nucleocapsid protein antibody (NovusBiologicals, Littleton, CO, USA) and with anti-SARS-CoV/SARS-CoV-2 (COVID-19) spike antibody [1A9] (GeneTex International Corporation, Alton Pkwy Irvine, CA, USA).

Immunohistochemical analysis was performed with anti-SARS nucleocapsid protein rabbit polyclonal antibody (Novus Biologicals), dilution 1:500, and anti-SARS-CoV/SARS-CoV-2 (COVID-19) spike mouse monoclonal antibody, dilution 1:250. An automated slide staining system (BenchMark ULTRA—Ventana Medical Systems, Inc.; Oro Valley, AZ, USA) was used. Three-micrometer thick tumor sections were dewaxed, pretreated with cell conditioner at 95 °C for 32 min with ULTRA CC1 ready-to-use solution (Ventana Medical Systems, Inc.), and then incubated with the aforementioned antibodies at 36 °C for 32 min. Antibody–antigen binding was detected by using the OptiView DAB IHC Detection Kit (Ventana Medical Systems, Inc.). Counterstaining was achieved with Hematoxylin II for 8 min and Bluing Reagent (Ventana Medical Systems, Inc.) for another 8 min, and then slides were dehydrated by sequential passages through increasing (from 70% to 100%) concentrations of ethanol and of xylene and mounted with coverslip. All of the immunostaining process was automated except manual antibody application and assembly of stained preparations. Staining positivity was shown as fine granular cytoplasmic stain.The presence of SARS-CoV-2 was also investigated by means of the Easy SARS-CoV-2 WE RT-PCR kit (Diatech Pharmacogenetics, Jesi, Italy) on formalin-fixed paraffin-embedded tissues. The assay is designed to target the viral nucleocapsid (N) and RNA-dependent RNA polymerase (RdRp) genes. Viral assays were run in duplicate using about 500 ng of total RNA per test. Samples were deemed positive when at least one of the N or RdRp genes was amplified.

## 3. Results

### 3.1. Case 1

A 74-year-old man with a medical history of arterial hypertension and ischemic heart disease in ASA treatment tested positive for SARS-CoV-2. Three days later, he presented dyspnea, severe desaturation, vomiting, and coma. He was intubated and admitted to the COVID Intensive Care Unit (ICU), after which he suffered acute abdominal distension. Blood tests revealed neutrophilic leukocytosis with PCT > 100 ng/mLand hyperamylasemia (504 U/L). Contrast-enhanced abdominal CT scan documented conglutinated jejunal and ileal loops, distal ileal “cockade”, large bowel distension, and initial signs of pneumatosis of the cecum. Laparotomy showed signs of ischemia of the terminal ileum and cecum. An ileocecal resection was made, with temporary abdominal closure through the negative pressure wound system. The peritoneal fluid, intraoperatively sampled, was not positive for SARS-CoV-2.

After 48 h, a scheduled second look was performed which showed wall congestion of the last 30 cm of the ileum, with unharmed mucosa. A terminal ileostomy and colonic mucous fistula were created, and the abdomen was closed. Twenty-fivedays later, the patient tested negative for SARS-CoV-2 and was discharged 10 days after surgery. A 50 cm long terminal ileum and cecum was analyzed at histology. The wall was diffusely edematous and the mucosa focally hemorrhagic with partially friable consistency. Microscopical examination of the ileum-colon showed widespread ischemic necrosis of the mucosal layer; the fibrin thrombi in the small vessels of the lamina propria and of the superficial submucosa are associated with pneumatosis. Mild acute perivascular inflammatory infiltration (neutrophils and lymphocytes), with foci of vasculitis, appears in the submucosal layer.

### 3.2. Case 2

A 69-year-old male patient with arterial hypertension and previous subdural hematoma was admitted to the Emergency Department after the onset of diffuse, severe abdominal pain. He tested positive for SARS-CoV-2. Contrast-enhanced CT scan of the abdomen documented pneumoperitoneum with “enlargement” of the distal portion of the duodenum, with air-fluid level in its context and suspicion of duodenal pseudodiverticulum perforation. Laparotomy showed multiple diverticula of the jejunum, one of which was perforated at about 40 cm from Treitz’s ligament. A 40 cm jejunal resection followed by anastomosis was performed. 

The patient was then transferred to the COVID Intensive Care Unit. The postoperative course was regular, and the patient was discharged on the 11thpostoperative day after a negative SARS-CoV-2 test. A short intestinal tract of 45 cm with some diverticula was analyzed. In correspondence to one of these, the wall appeared thickened, with evident emphysema of the perivisceral and subserosal adipose tissue. Microscopic examination showed edema of the intestinal villous and intense lymphoplasmacytic infiltrate in the lamina propria. Fibrin thrombi in the small vessels of the mucosa and in the submucosal layer with pneumatosis of the submucosal adipose tissue were evident. 

### 3.3. Case 3

**Figure 1:**
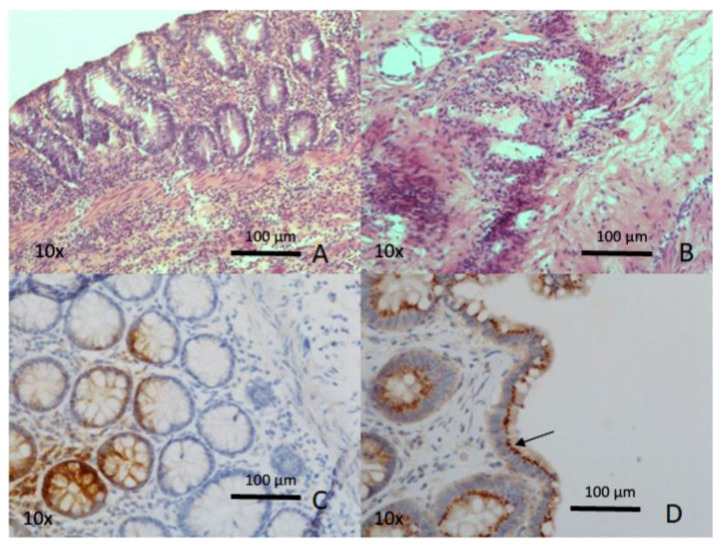
(Hematoxylin-eosin staining; original magnification, 10×) Photomicrograph of the small intestine shows widespread inflammatory infiltrate in the lamina propria and in the submucosal layer (**A**) as well as perivascular (**B**). Immunohistochemistry, anti-SARS-CoV-2 spike glycoprotein (**C**), and SARS nucleocapsid protein antibody (**D**): extensive and well-defined cytoplasmic, supranuclear (arrow) immunostaining in the mucosal epithelial cells.

A 77-year-old male was hospitalized for COVID-19-related respiratory problems. His past medical history was positive for smoking, arterial hypertension, type 2 diabetes mellitus, chronic obstructive pulmonary disease, chronic ischemic heart disease, previous pulmonary embolism, and ischemic stroke. He was submitted to Hartmann’s resection for sigmoid diverticular perforation in 2014. Following the onset of peritonitis and CT evidence of diffuse pneumoperitoneum, the patient underwent laparotomy. A millimetric perforation was identified in the context of ischemia of the ascending colon. Right hemicolectomy with terminal ileostomy and mucous fistula were performed. The intraoperative sample of peritoneal fluid was negative for SARS-CoV-2. Having tested negative for COVID-19, the patient was discharged two weeks after surgery. A 7 cm long terminal ileum and a 29 cm long ascending colon were resected. The wall was thinned along the entire tract, and the mucosal profile was attenuated. Microscopic examination showed focal epithelial erosions, acute inflammation, and micro-hemorrhage of the lamina propria, with foci of vasculitis. Pneumatosis was evident in the submucosal layer (Figure 1).

### 3.4. Case 4

A 75-year-old male patient presented diffuse acute abdominal pain. Consequently, he came to the Emergency Department, where he tested positive for SARS-CoV-2. In anamnesis, he had double myocardial infarction with endovascular double stenting placement, atrial fibrillation under oral anticoagulation therapy (Rivaroxaban), and gastric ulcer. His surgical history was positive for appendectomy, excision of pulmonary tuberculoma, and multiple orthopedic operations. The contrast-enhanced thoraco-abdominal CT scan revealed a small, covered perforation at the jejunum-ileal passage, with presence of air in the context of the mesentery, and incidental interstitial pneumonia. Laparotomy was carried out: a purulent effusion with perforation of a jejunal loop at about 30 cm from Treitz’s level was identified. A resection anastomosis was performed. SARS-CoV-2 was not found in the peritoneal fluid. 

The postoperative course was complicated by a superficial infection of the surgical site, successfully treated with debridement. He was discharged 46 days after surgery. Two tracts of the small intestine, respectively 13 cm and 7.5 cm long, were excised. In the longer tract there were some diverticula, one of which appeared perforated and with fibrin stratification. Microscopical examination showed widespread mucosal and submucosal hemorrhage with acute necrotizing inflammation around the diverticula and numerous fibrin thrombi, with vasculitis in the small-medium size vessels. 

### 3.5. Case 5

A 72-year-old male patient, smoker, affected by post-ischemic dilated heart disease, permanent atrial fibrillation, arterial hypertension, dyslipidemia, and chronic obstructive pulmonary disease, was hospitalized for COVID-19-related interstitial pneumonia. He was admitted to hospital with hemoptysis, for which he underwent an upper endoscopy. A clot identified in the duodenum was treated by endoscopic hemostasis with adrenaline injection. A contrast-enhanced CT scan of the abdomen documented active arterial endoluminal bleeding in the third tract of the duodenum. A further endoscopic hemostasis was warranted. However, owing to the onset of hematemesis and abdominal pain, the patient received another CT scan with contrast which revealed free abdominal fluid and isolated air microbubbles at the level of the mesenteric fan, at the left median-paramedian site, and at the level of the duodenal-jejunal flexure. Therefore, an angioembolization of the gastroduodenal artery branches was accomplished successfully. Two days later, recurrence of active bleeding made it necessary to perform exploratory laparotomy with a bleeding ulcer of the third duodenal portion and jejunal infarction. Thus, a suture of the duodenal ulcer and multiple jejunal resections were carried out. The abdomen was packed, and a damage control strategy was adopted. The following day, a recurrence of the hemoperitoneum occurred which was caused by diffuse bleeding sources in the right retroperitoneal area at intra-abdominal exploration. Therefore, accurate hemostasis, abdominal packing, and skin closure were carried out. At a third look, a duodenal fistula with abundant bile leakage and active bleeding in the periduodenal region were identified. Gastroenteroanastomosis, cholecystectomy with placement of transcystic biliary drain, and intestinal patch repair of the duodenal fistula were performed. During the following postoperative course, the patient presented sepsis and generalized peritonitis. Thus, a new laparotomy was performed; partial dehiscence of the patch and duodenal sinking at Treitz’s level, with diffuse purulent peritonitis, were found, so that peritoneal toilet, suture of the dehiscence, and Foley catheter duodenostomy were accomplished. However, on the 10th postoperative day, the patient died on account of a septic shock. The samples consisted of two small intestine tracts, respectively, 4.5 cm and 50 cm long. The wall was uniformly dilated, and the mucosal profile was attenuated with fibrin stratification.

Microscopic examination showed widespread ischemic necrosis and ulceration in the adipose tissue and in the mucosal and muscular layers. Acute inflammatory infiltrate, formed by neutrophils and lymphocytes, expanded full thickness skin graft. Fibrin thrombi in the small vessels of the lamina propria and of the submucosal layer were present. 

The main anamnestic and clinical features of each patient are summarized in Table 1. Surgical interventions, outcomes, and histopathological findings are reported in Table 2.

### 3.6. Immunohistochemical and Molecular SARS-CoV-2 Detection in Paraffin-Embedded Tissue

All cases were analyzed for SARS-CoV-2 detection in paraffin-embedded tissue for immunohistochemical and molecular analysis. Both analyses were successfully performed in all cases. Although immunohistochemical positivity with weak intensity was observed in four out of five cases, only *case 3* was strongly positive both on immunohistochemistry and on reverse transcription polymerase chain reaction (RT-PCR) analysis (Table 2).

## 4. Discussion

Gastrointestinal manifestations were described shortly after the SARS-CoV-2 outbreak as uncommon and self-limited symptoms of COVID-19 infection [12,13]. To date, an increasing number of studies have underlined digestive system involvement in the SARS-CoV-2 syndrome and the possibility of related high-risk complications [14]. SARS-CoV-2 can cause direct injury of the gastrointestinal mucosa through its attachment to the angiotensin-converting enzyme receptor (ACE2), which is abundantly expressed on enterocytes [12,15], or by disruption of the normal colonic gut flora or by bowel ischemia resulting from thromboembolic complications [16]. Although the exact pathogenesis of COVID-19-associated hyper-coagulopathy remains unclear [17], COVID-19 has been demonstrated to be linked to coagulopathy [13,18].

Our series shows the presence of all male patients. Interestingly, this finding is in accordance with recent systematic reviews of the literature about COVID-19 patients with acute intestinal ischemia: Fransvea et al. [19] analyzed 25 articles and reported 32 patients, among which 23 were male (72%); another systematic review of 36 articles by Serban et al. [20] showed a 61.5% incidence of male sex on 89 enrolled patients, confirming the male-to-female predominance.

Acomprehensive assessment, including clinical parameters and laboratorial (white blood count and C-reactive protein) and radiological data, should be made to evaluate the possible presence of a SARS-CoV-2 gastrointestinal infection and to monitor and evaluate the severity and prognosis of the disease [21,22]. Abdominal radiological findings are not specific and have a poor pathological correlation in the literature [23]. Abdominal CT scans are reported to be positive in 20% of analyzed COVID-19 patients, intestinal distension and small bowel thickening being the most prevalent findings [22].

Gastrointestinal disorders are mostly self-limiting, but severe complications may occur, among which intestinal ischemia is the most fearsome [24]. Farina et al. [25] reported the case of a patient with clinical signs, symptoms, and chest CT findings consistent with COVID-19 but who tested negative at nasopharyngeal swab. Contrast-enhanced CT of the abdomen showed acute small bowel hypoperfusion due to acute superior mesenteric artery (SMA) thrombosis. The patient was declared inoperable and passed away 2 days after admission, despite the medical treatments.

Cheung et al. [17] described the case of a patient affected by nausea, generalized abdominal pain, and diarrhea, without fever or respiratory symptoms, radiologically diagnosed with COVID-19 pneumonia, and treated with medical therapy. One week after discharge, owing to the recurrence of worsening diffused abdominal pain, a CT scan was repeated which revealed intestinal ischemia caused by acute SMA thrombosis. He was successfully treated with thromboembolectomy and resection of the ischemic small bowel.

These reports further urge clinicians to monitor all suspected/confirmed COVID-19 patients for the possible presence of a SARS-CoV-2 intestinal infection to identify early any abdominal change suggestive of ischemic complications.

During follow-up, abdominal CT scan (in case of unexplained worsening status), and/or laboratory examination suggestive for intestinal ischemia (even in case of negative clinical examination of the abdomen) are mandatory [2].

Bhayana et al. [10] reviewed the abdominal imaging studies of 134 COVID-19 patients. They identified bowel findings of pneumatosis or portal venous gas in 20% of CT scans of ICU patients and correlated them with surgical reports in four cases; they showed that these signs were intraoperatively correlated with frank bowel infarction (*n* = 2) and with the presence of an unusual yellow discoloration of the bowel (*n* = 3).

The limited case studiesconcerning CT findings in SARS-CoV-2 gastrointestinal infection and the poor knowledge about their prognosis make the decision as to whether to operate on these frail patients a challenge [2]. Stable and asymptomatic cases of COVID-19 patients suspected of intestinal ischemia with no signs of systematic deterioration could be managed conservatively by medical therapy and elemental diet. Kielty et al. [26] described the case of a 47-year-old man affected by COVID-19 with CT findings of diffuse small-bowel distension, widespread pneumatosis, circumferential mural thickening, free fluid, and mesenteric free air and portal venous gas. The patient was successfully treated by infusion of unfractioned heparin. Aiello et al. [27] reported the case of a 73-year-old man with COVID-19 pneumonia under immunosuppressive and steroid therapy for previous renal transplant with CT findings of intestinal pneumatosis but with no corresponding abdominal pain. The patient was conservatively managed with a course of oral metronidazole and discharged without any GI symptoms.Similarly, Meini et al. [28] described the case of a 44-year-old man with incidental CT finding of pneumatosis involving the cecum and the right colon. He experienced a complete radiological resolution after two weeks of ciprofloxacin and metronidazole administration.

Surgery is required in critically ill or unstable patients, as well as in cases of perforation, peritonitis, abdominal sepsis, and failed conservative management. Surgical intervention is also warranted if the patient’s condition rapidly deteriorates. Other indications for operation include a combination of age (>60 years), leukocytosis, elevated lactic acid, and radiological finding of portal venous gas [28]. In our series, the reason for an exploratory laparotomy was the radiological evidence of an intestinal perforation in most cases (#2, 3, 4, and 5) and the radiological presence of parietal pneumatosis in one case (#1). In all cases, the ischemic tract was resected, and the type of reconstruction was decided according to a patient’s general conditions, site of ischemia, viability of the residual bowel, and grade of abdominal contamination.

If the viability of the bowel remains doubtful, a second-look procedure should be scheduled and performed. Bowel continuity restoration with anastomosis should be carried out only in stable patients without abdominal sepsis/septic shock. Among our patients, primary anastomosis was performed in two cases of ischemia involving the jejunum; in one case of ischemia of the ascending bowel, colonic mucous fistula and permanent ileostomy were created; in the other two cases, which consisted in an ileocecal ischemia and a jejunal infarction with duodenal ulceration, the abdomen was left open, and a laparostomy was packed. Of the two laparostomies, the first was followed by a second look with terminal ileostomy and colonic mucous fistula creation, while the second experienced a postoperative course complicated by the spontaneous formation of a duodenal fistula that led to the patient’s exitus for septic shock.

COVID-19 may also appear with only gastrointestinal involvement without respiratory symptoms [4]. Zamboni et al. [29] reported three cases of asymptomatic patients tested negative for COVID-19 and presenting with signs and CT findings of acute bowel ischemia of thrombotic origin and all treated with emergency intestinal resections. Interestingly, the authors found strong SARS-CoV-2 positivity at immunohistochemical analysis of the resected tissues. Norsa et al. [30] reported the case of a man with onset of abdominal pain and bilious vomiting who tested negative for SARS-CoV-2 in nasopharyngeal swab and at chest CT, with abdominal CT findings highly suggestive ofsmall bowel ischemia. The patient underwent small intestine resection but died of refractory septic shock within few hours from surgery. The authors interestingly demonstrated the presence of SARS-CoV-2 spike protein mRNA through RNA ISH assay in the intestinal mucosa of the surgical specimen.

In our series, we found strong COVID-19 positivity only in one resected specimen that was confirmed by both immunohistochemistry and molecular analysis. However, the histopathological evidence of endothelial inflammation in the submucosal vessels that all of our patients showed in the intestinal tract suggests a microvascular small-bowel injury comparable to the findings in the literature that describe the small blood vessels of COVID-19 lungs. Support for inflammation-mediated tissue damage is provided by the presence of infiltrating plasma cells and lymphocytes and of interstitial edema in the lamina propria of the small intestine and in the colon of patients. The presence of the virus in the tissue was found in only one patient. However, these data have already been reported in the literature. Recently, from a cohort of 95 COVID-19 patients, six individuals underwent endoscopic examination, and two harbored the SARS-CoV-2 RNA in the esophagus, stomach, duodenum, and rectum: these same had the most severe form of viral infection, suggesting a correlation between the severity of symptoms and the location of the virus in the gastrointestinal tissue [31].

We are aware of the limits of this study, mainly due to its retrospective observational nature and to the small number of cases presented. Thus, further studies on a larger number of samples are needed to explain the etiology of intestinal ischemia in COVID-19 patients. The observed histological alterations in the bowel are not different from those found in other organs and tissues targeted by the virus and widely described in the literature. This suggests a similar mechanism of action of the virus that has proven to be linked to the presence of specific cellular receptors (ACE2, TMPRSS2) [32]. Therefore, an evaluation of the expression of these receptors on intestinal tissue would be interesting, as it could be postulated that higher expression correlates with higher severity of intestinal damage.

## 5. Conclusions

COVID-19 may present with gastrointestinal symptoms associated or not with respiratory and systemic clinical manifestations. Surgery is mandatory in case of perforation, peritonitis, abdominal sepsis, and failed conservative management. It is not clear whether the etiology of intestinal ischemia may be directly related to the viral replication or the consequence of hyper-coagulability following SARS-CoV-2 infection. Further studies with larger sample sizes are thus required.

## Figures and Tables

**Table 1:** Characteristics of COVID-19 patients with intestinal ischemia.

	**CASE 1**	**CASE 2**	**CASE 3**	**CASE 4**	**CASE 5**
**Age** **(years)**	74	69	77	75	72
**Gender** **(male/female)**	M	M	M	M	M
**Comorbidities**	Arterial hypertension, ischemic heart disease in treatment with ASA, HCV+	Arterial hypertension, previous subdural hematoma	Arterial hypertension, smoking, type 2DM, COPD, chronic ischemic heart disease, previous pulmonary embolism, and ischemic stroke	Double acute myocardial ischemia treated with double endovascular stenting, atrial fibrillation under oral anticoagulation therapy, and gastric ulcer	Arterial hypertension, post-ischemic dilated heart disease, COPD permanent atrial fibrillation, dyslipidemia
**Previous abdominal surgery**	NO	NO	YES (Hartmann’s resection for sigmoid diverticular perforation)	YES (Appendectomy)	NO
**COVID-19 respiratory infection (yes/not)**	YES	YES	YES	YES	YES
**Clinical presentation**	Acute abdominal distention	Diffuse severe abdominal pain	Acute abdominal pain with peritonitis	Diffuse acute abdominal pain	Hemoptysis
**CT findings**	Initial sign of pneumatosis of the cecum	Pneumoperitoneum with “enlargement” of the distal portion of the duodenum	Diffuse pneumoperitoneum	Small, covered perforation at the jejunum-ileal passage, with pneumatosis of the mesentery	Peritoneal fluid, initial pneumatosis of the mesenteric fan and of the duodenal-jejunal flexure wall

**Table 2:** Surgical interventions, outcomes, and histopathological findings of treated patients.

	CASE 1	CASE 2	CASE 3	CASE 4	CASE 5
**Surgery**	Laparotomic ileocecal resection with temporary abdominal closure (NPWT) Second look: terminal ileostomy, colonic mucous fistula, and definitive abdominal wall closure	Laparotomic jejunal resection of perforated diverticulum and anastomosis	Laparotomic right hemicolectomy with terminal ileostomy and mucous fistula	Laparotomic resection of perforated jejunal loop at about 30 cm from Treitz’s level and anastomosis	Laparotomic suture of duodenal ulcer, multiple jejunal resections and packing Second look: hemostasis of retroperitoneal bleeding, abdominal packing, and skin closure III look: gastroentero-anastomosis, cholecystectomy with placement of transcystic biliary drain, intestinal patch repair of duodenal fistula, and abdominal wall closure. Peritoneal toilet, suture of the dehiscence of the patch at Treitz’s level, and Foley catheter duodenostomy
**Complications**	Respiratory complications, tracheostomy	NO	NO	NO	Septic shock
**Mortality**	NO	NO	NO	NO	YES
**Histology**	Mucosal ischemic necrosis with fibrin thrombi of small vessel and pneumatosis	Mucosal edema with fibrin thrombi of small vessel and pneumatosis	Mucosal erosion and microhemorrage with fibrin thrombi of small vessel and pneumatosis	Mucosal edema and microhemorrage with fibrin thrombi of small vessel	Mucosal widespread ischemic necrosis and ulceration with fibrin thrombi of small vessel
**PCR per SARS-CoV-2 (specimen)**	negative	negative	*positive*	negative	negative

## Data Availability

The data presented in this study are available on request from the corresponding author. The data are not publicly available due to privacy reasons.
